# High Content Analysis Across Signaling Modulation Treatments for Subcellular Target Identification Reveals Heterogeneity in Cellular Response

**DOI:** 10.3389/fcell.2020.594750

**Published:** 2021-01-07

**Authors:** Sayan Biswas

**Affiliations:** Department of Biochemistry, School of Life Sciences, University of Hyderabad, Hyderabad, India

**Keywords:** phenotypic similarity, signaling modulation, cellular and organelle behavior, predictive modeling, heterogeneity in responses, mechanism of action, high content imaging screen

## Abstract

Cellular phenotypes on bioactive compound treatment are a result of the downstream targets of the respective treatment. Here, a computational approach is taken for downstream subcellular target identification to understand the basis of the cellular response. This response is a readout of cellular phenotypes captured from cell-painting-based light microscopy images. The readouts are morphological profiles measured simultaneously from multiple cellular organelles. Cellular profiles generated from roughly 270 diverse treatments on bone cancer cell line form the high content screen used in this study. Phenotypic diversity across these treatments is demonstrated, depending on the image-based phenotypic profiles. Furthermore, the impact of the treatments on specific organelles and associated organelle sensitivities are determined. This revealed that endoplasmic reticulum has a higher likelihood of being targeted. Employing multivariate regression overall cellular response is predicted based on fewer organelle responses. This prediction model is validated against 1,000 new candidate compounds. Different compounds despite driving specific modulation outcomes elicit a varying effect on cellular integrity. Strikingly, this confirms that phenotypic responses are not conserved that enables quantification of signaling heterogeneity. Agonist-antagonist signaling pairs demonstrate switch of the targets in the cascades hinting toward evidence of signaling plasticity. Quantitative analysis of the screen has enabled the identification of these underlying signatures. Together, these image-based profiling approaches can be employed for target identification in drug and diseased states and understand the hallmark of cellular response.

## 1. Introduction

Measurement of biological activity upon small molecule-based treatment has the potential to illustrate the mechanisms of action by comparing it with profiles of known compounds (Hughes et al., 2000; Lamb et al., 2006; Feng et al., 2009). These measurements from high-throughput target-directed screens have been widely used for their potential application in drug discovery through unbiased testing of several million compounds per screen (Macarron et al., 2011). Phenotypic screening has also been proposed for efficient assessment of drug candidate testing in biological systems (Lee et al., 2012; Futamura et al., 2013). These approaches are facilitated by quantitative microscopy, widely used in pharmaceutical and academic labs, since it provides a versatile and powerful readout for precise cellular measurements and identifying cellular states (Carpenter, 2007; Futamura et al., 2013). The principle of phenotypic profiling is based on summarizing multiparametric, feature-based analysis of cellular phenotypes of each sample so that sample similarities are reflected on similarities between profiles (Wagner and Clemons, 2009). Transcript expression and proteomics profiling serve as established biological readouts (Hughes et al., 2016; Szalai et al., 2019). In comparison, image-based profiling is cost effective and flexible for scaling between medium and high throughput with relative ease, alongside providing phenotypic details at single-cell resolution (Ljosa et al., 2013). Although image-based screens aim to score samples with respect to one or a few known phenotypes, profiling experiments aim to capture phenotypes not known in advance, using a variety of subtle cellular responses and widely used as predictive models (Ljosa et al., 2013; Kandaswamy et al., 2016; Steigele et al., 2020). A mechanism of action (MoA) usually refers to biochemical interaction through which the drug acts to induce pharmacological effect and phenotypic changes (Kandaswamy et al., 2016), which can be studied based on the phenotype.

This potent research paradigm has been employed over the past few decades by the pharmaceutical and biotechnology sectors (Moffat et al., 2014, 2017). Drug discovery through cell systems biology could significantly reduce the time and cost of new drug development (Butcher, 2007). Automated high-content microscopy imaging and image analysis methods offer an efficient alternative to the traditional target-directed screening approach (Lang et al., 2006; Simm et al., 2018; Nyffeler et al., 2020). This allows researchers to study the cellular phenotype response on molecular perturbations irrespective of putative target activity (Tanaka et al., 2005; Low et al., 2008). Computational application of such methods to study cellular response relies heavily on active measurements that can capture a spectrum of phenotype. Assays with multiple fluorescent markers enable to capture quantitative profiles in high throughput. These methods provide an unbiased approach to study cell states associated with chemical perturbation and disease state to support future probe discovery. Such cellular assays show the value of phenotypic profiling to assist not only in the identification of cellular activity and but also to develop an understanding to elucidate the MoA for drugs whose mode of action or primary targets are unknown (Loo et al., 2007; Young et al., 2008; Caie et al., 2010; Breinig et al., 2015). The ability to identify the targets of candidate molecules in a screen can help overcome one of the bottlenecks to establish it as a drug. Although experimental approaches for target identification in a screen could be labor, resource, and time intensive; computational approaches substantially reduce the work and resource requirement for favorable application (Perlman et al., 2011; Chen et al., 2016; Madhukar et al., 2019). However, a key challenge in the field is the identification of the sub-cellular effects caused by the treatment and also understand the basis of the cellular responses.

In this report, it is aimed to identify the downstream organelle targets by using computation approaches on the “cell-painting” assay screen. A quantitative understanding of the heterogeneous cellular responses in the treatment screen based on the subtle changes in cellular phenotype profiles is demonstrated. This approach fosters the possibility for a quantitative examination of the responses induced by selective pharmacologic agents across cancerous cells. Subsequent analysis demonstrated the role of conserved and differential signatures in the diverse organelle behavior in the multifaceted cellular response. This interconnected dependence is exploited for developing models to predict the overall cellular response based on specific organelle response. Further advancement is achieved through fine quantification, which elucidated the varying cellular response even when the treatment outcome is conserved hinting toward signaling heterogeneity.

## 2. Methods

### 2.1. Dataset

Here the “Cell-Painting” (Bray et al., 2016) assay as documented in BBBC022v1 (Gustafsdottir et al., 2013) has been used. This is publicly available from the Broad Bioimage Benchmark Collection (Ljosa et al., 2012) and is one of the widely used dataset in the field. The raw data have been downloaded as documented in an earlier published report (Gustafsdottir et al., 2013) (from http://www.broadinstitute.org/pubs/gustafsdottir_plosone_2013/). In this dataset, bone carcinoma U2OS cells are imaged on treatment with multiple bioactive compounds. The cells are fluorescently labeled to follow the components: Golgi, endoplasmic reticulum (ER), nuclei (Hoechst), nucleoli (Syto), and mitochondria (Mito). The bio-active compounds are chemical perturbations and are referred to have specific BroadID. To specifically annotate the treatment compounds with the relevance of the induced phenotype or the respective MoA, the “ground truth” of the image data (Corsello et al., 2017) made available as part of the BBBC036 (Bray et al., 2017) from the Broad Bioimage Benchmark Collection (Ljosa et al., 2012) has been used. This allowed ~270 MoAs to be successfully annotated (Supplementary Table 1), which forms the working dataset for this study. There were roughly 1,000 compounds (or BroadIDs) (Supplementary Table 2) in the dataset for which MoA could not located based on BBBC036 file. These compounds have been used as test compounds for the prediction model developed as described in **Figure 3C**.

Cell Profiler (Carpenter et al., 2006) pipeline has been engineered (Gustafsdottir et al., 2013) to extract rich quantitative features (Supplementary Table 3) at single-cell resolution from the light microscopy images. For locating the origin of features, the occurrences of acronym Golgi, ER, Hoechst, Syto, and Mito in feature labels are used for feature originating from Golgi, endoplasmic reticulum, nucleus, nucleoli, and mitochondria, respectively (Supplementary Table 3). These features are used to report the dynamics associated with the specific organelle. An example of the dataset with 100 cells is illustrated in Supplementary Table 5.

### 2.2. Analysis

The script developed for the analysis presented in this study is done using MatLab. Required details of the parameters have been enlisted in the respective section 3.

**Figure 1:**
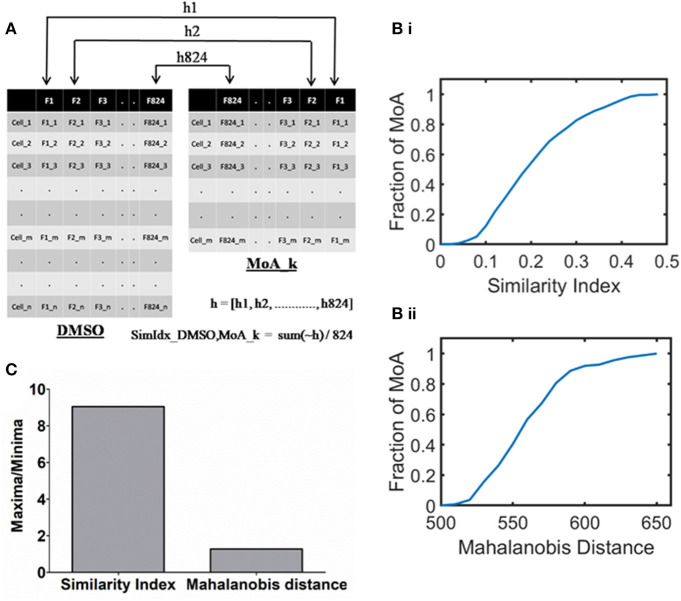
Quantifying the cellular response upon mechanism of action inducing treatment with respect to DMSO. **(A)** A schematic outlining the method of Similarity Index calculation between DMSO (with n cells) and kth MoA (with m cells). Note that 824 single cell features (represented as columns) form the phenotypic profiles. **(B)** Cumulative histogram of the MoAs in the annotated dataset based on (i) SimIdx and (ii) Mahalanobis distance, demonstrating the varied response among the different signaling modulation treatment. **(C)** Quantification of the dynamic range (maxima by minima) calculated for each of the metric.

- Similarity index: Two-sample *t*-test has been performed at a 5% significance level. Two tail test has been performed. It is performed to see if a feature has changed significantly in an MoA with respect to the same feature for DMSO treatment (Figure 1A). Two sample test is performed to compare between DMSO and the MoA for which hypothesis testing is performed.
- Mahalanobis distance: This computes the distance in multivariate space between a point and distribution. The features of DMSO form the distribution and each cell at MoA forms a point. This measure is often used for outlier filtering in biomedical multivariate data (Laurikkala et al., 2000). Similarly, in this case prior to plotting histogram (Figure 1Bii), outlier detection has been performed.
- Multivariate regression: This has been performed using *fitlm* (Holland and Welsch, 1977). All possible combinations of two and three organelles have been used as predictor variables. The output (response) variable is the overall cellular response. The target is to regression model the organelle response (all combinations of two and three variables) to predict overall cellular response.
- Goodness of prediction: The multivariate regression models are used to estimate the goodness of prediction. For the testing, the test set contains data from 1,000 of new compounds. The predictor values are derived from the test dataset and thereby response value is estimated based on the regression model. To check the goodness of prediction, the estimated response value and actual response value are compared. This error is used to determine sum of squares due to error (SSE) and the total sum of squares (SST). R square value is calculated as 1−*SSE*/*SST*. A good prediction would mean low error, which means a higher R square value.

## 3. Results

### 3.1. Quantification of the Cellular Response to Signaling Modulation

Signaling modulation through chemical agent treatment causes a spectrum of phenotypic responses (Kitano, 2002; Wawer et al., 2014) in the cells. These responses or the cellular integrity changes, as a result of the treatment, could be captured from the cellular morphology with the help of quantitative microscopy. The publicly available dataset of “cell painting” extracts these morphological phenotypic profiles from various cellular compartments through microscopy and image quantification at the single-cell resolution. Furthermore, the outcome of the signaling modulation (or the MoA) that the respective compound induces has also been annotated. Hence to study the cellular responses and effect of the treatments, the cell-painting assay dataset has been chosen. Phenotypic profiling summarizes cellular phenotypes upon the treatment, allowing the study of similarities between treatment by studying the profiles (Wagner and Clemons, 2009). The DMSO-treated cells are also profiled to extract the rich quantitative features. Here DMSO serves as the control (Galvao et al., 2014) for the chemical agent treatment. Therefore, to systematically address the cellular response due to the treatment, the similarity in the morphological features (Supplementary Table 3) between the treatment and DMSO is assessed.

To quantify this, the significance is tested between respective features of MoA with that of DMSO through p-value as illustrated in Figure 1A. Here *h* is a binary array that contains 824 elements, where *x*^*th*^ element signifies whether *x*^*th*^ feature is similar [0] or not [1] between the MoA treatment and DMSO. The parameter of similarity index (SimIdx) is then quantified for the MoA based on the fraction of similar features (number of zeros in the “h” array) between DMSO and the MoA. Thus, SimIdx is calculated to depict the similarity between the MoA inducing treatment and DMSO in terms of the phenotypic features, which can have a value between 0 and 1 signifying minimum and maximum similarity, respectively.

This process is then iterated across all the MoA treatments in the working dataset. Thus, a cumulative histogram is plotted to show the distribution of SimIdx calculated across all the MoA inducing treatments as shown in Figure 1Bi. While a fraction of MoAs has SimIdx close to 0, a significant fraction has it close to 0.5, the highest end of the curve. Therefore, the dynamic band of SimIdx helped to identify the spectrum of responses different MoA poses with respect to DMSO in terms of phenotypic similarity.

To compare this observation, the established method of Mahalanobis distance is also used to determine the phenotypic difference MoA exhibits with that of DMSO. Briefly, this metric helps capture distances in a multivariate feature space. A lesser value of Mahalanobis distance would signify lesser difference between the phenotypes of MoA and DMSO and vice versa. As mentioned earlier, a cumulative histogram is obtained for the Mahalanobis distance metric calculated between the MoAs in the working dataset and DMSO (Figure 1Bii). This affirms the varying response the MoAs contained in the dataset exhibit.

Based on the histograms (Figure 1B), dynamic range is derived by calculating (dividing maxima by minima) for each of the metrics as indicated in Figure 1C. The same working dataset of MoA treatments has been calibrated with both the metric but the SimIdx resolves the innate differential response better than Mahalanobis distance as indicated from the dynamic range. Put together, these two quantitative measures reflect the differential response various MoA exhibits with respect to DMSO.

### 3.2. Impact on the Organelle Induced by the Treatment

**Figure 2:**
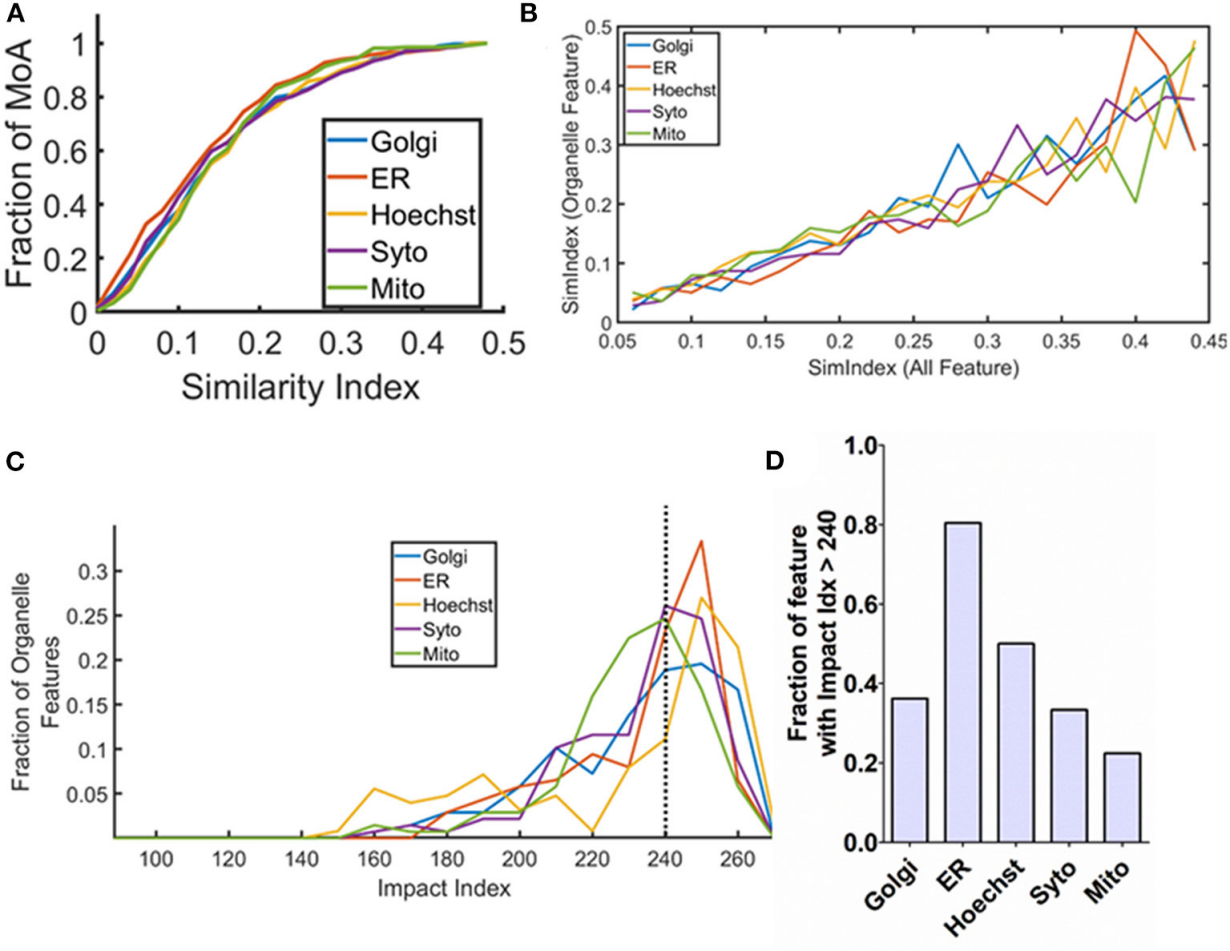
Specific organelle-based responses on the signaling modulation treatment. **(A)** Cumulative histogram of the similarity index calculated based on the organelle features based on the mechanism of actions (MoAs) in the annotated dataset. **(B)** Co-relation curve showing overall SimIdx and organelle-specific SimIdx. The graph is obtained using all the MoAs in the dataset. **(C)** Representation of impact index of the organelle features in the form of a histogram. **(D)** Bar plot showing the fraction of organelle features that has impact index value of more than 240.

Recognition of sub-cellular compartments affected by a modulation treatment is critical to identify the respective treatment's downstream target. Therefore, the next aim is to monitor the impact on specific organelles. For this, the metric of SimIdx is utilized that enables to compare the phenotypic changes caused by the signaling modulation treatments. The cell painting assay facilitates to address this since it allows simultaneous monitoring of multiple organelles—ER, nucleus (Hoechst), nuclelous (Syto), mitochondria (Mito), and Golgi (Golgi) through targeted fluorescent probes. Thereby the features that originate from the specific organelle targeted fluorescent labels are explicitly identified (Supplementary Table 3) among all the features. By specifically comparing the organelle features, the organelle SimIdx has been determined. This calculation paved the understanding of how particular organelle integrity changes upon a signaling modulation treatment. Thus for every MoA treatment, it resulted in five values of organelle SimIdx, one for each of the organelle. These organelle SimIdx values account for changes in that specific organelle integrity due to the signaling modulation treatment. The distribution of this parameter for all the MoA inducing treatments available in the dataset is plotted as a cumulative histogram in Figure 2A. The graph shows that this parameter encompasses a diverse range, revealing the variation in the organelle response as well. To investigate this response, the existence of any coupling between the integrity changes in the overall cell and those of specific organelle for respective signaling modulations treatment is examined. Based on the diverse MoA inducing treatments, the plot of specific organelle SimIdx vs. all feature SimIdx is illustrated in Figure 2B. The trend shows that changes in the cellular response are reflected as conserved changes in the organelle response. The subtle changes of organelle phenotype are conserved with respect to overall cellular integrity changes, which elucidates an underlying conserved signature in the cellular responses.

As mentioned earlier, each organelle phenotype is profiled based on more than 100 features (Supplementary Table 3). It is then assessed; each of these features is affected by how many of the treatments? To extract this information, Impact Index (ImpIdx) is quantified for each of the features. First, a binary array *SimVal* is determined for each feature (in Equation 2 it is represented for 1*st* feature or *f*1), which is an array of 270 elements (number of MoAs in this study). The *i*^*th*^ element of this binary array signifies if *f*1 has been affected [1] by the *i*^*th*^ MoA inducing treatment or not [0]. ImpIdx value for feature *f*1 is then calculated by adding all elements of *SimVal*_*f*1_ as per Equation (2). For every feature, the ImpIdx value would be between zero and the total number of MoA inducing element in the dataset where the extremes would mean that the feature has been impacted significantly for none or all of the MoA inducing treatment. Thus, this parameter is directly proportional to the likelihood estimate of the feature to be impacted upon a treatment.

(1) $$\begin{array}{r} SimVal_{f1}=\left[0,1,1,0,........,1,0\right]\left(\sim 270Elements\right) \end{array}$$

(2) $$\begin{array}{r} ImpIdx_{f1}=\sum \left(SimVal_{f1}\right) \end{array}$$

In this way, ImpIdx values are obtained for all the 824 features. Using this metric, the aim is to assess which organelles have a higher chance of being impacted downstream of the treatments. To pursue this, ImpIdx from specific organelle features are then collated. The distribution of organelle ImpIdx is shown in Figure 2C. Based on this distribution, a finer quantification is performed to identify the characteristic downstream target organelle. This is identified based on the fraction of organelle features that demonstrate ImpIdx of greater than 240 (roughly 90% of its maximum possible value, 0.9 × 270 = 243). These organelle fractions are represented in Figure 2D, which shows that 80% of ER features express quite high ImpIdx. These revealed that ER is a downstream target for most of the drug treatments performed in this study. In contrast, the mitochondria features express comparatively lesser impact, likely signifying the less pronounced effect by these treatment molecules on mitochondria. Put together, this analysis not only showed coupling between the overall cellular and specific organelle response but also established organelle signatures based on its likelihood of being affected upon treatment.

### 3.3. Sensitivity Detection and Prediction of Overall Cellular Response

**Figure 3:**
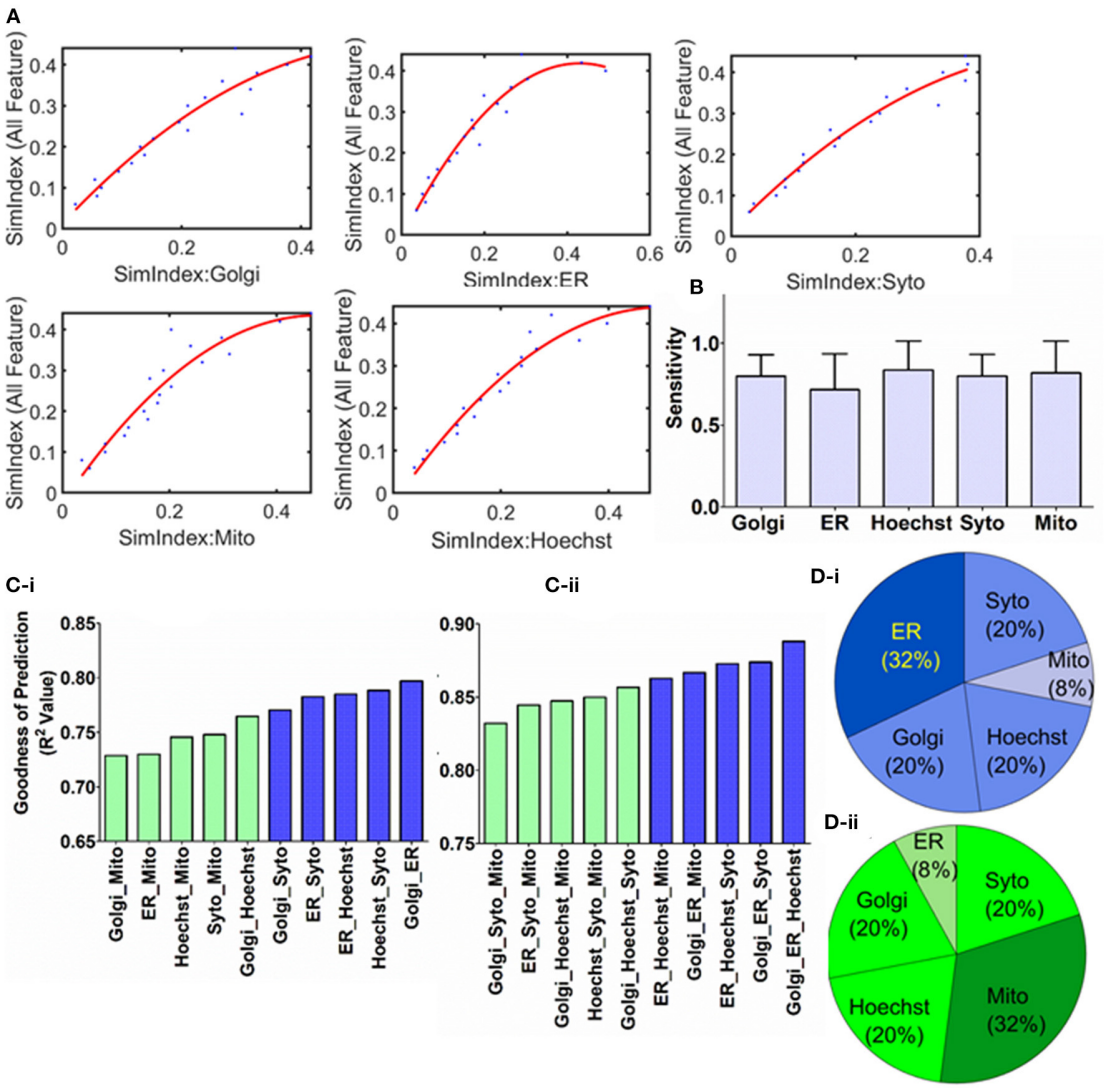
Characterizing the correlation and regression modeling. **(A)** Quadratic polynomial fitted with the organelle response as the independent variable and cellular response as the dependent variable. **(B)** Sensitivity of the cellular response to the organelle response has been quantified through the first-order derivative of the polynomial fitted earlier. **(C)** Multivariate regression analysis is performed with organelle response as independent variable, while the cellular response is the dependent variable. Using this regression model, cellular response is predicted for over 1,000 new compounds. Based on the error between actual and estimated values goodness of prediction is quantified by using R squared values. These calculations are performed for all possible combinations of (i) two and (ii) three organelles as independent variable. Blue and green indicate the combinations that yielded the top 5 and bottom 5 goodness of prediction, respectively, in each case. **(D)** The occurrence of each organelle in (i) top 5 and (ii) bottom 5 models as per goodness of prediction is plotted as pie chart.

Sensitivity could be one of the hallmarks of biological response and can be useful to extract a direct relationship between the pharmacological agent treatment and resultant downstream response. To address this, first, the correlation curves (Figure 2B) are characterized by regression modeling. These regression models are developed separately for each of the organelles and depicted in Figure 3A (Supplementary Table 6). Next, the first-order derivative is computed on these curves to extract the sensitivity of the organelle response due to the treatment. The resultant sensitivities are shown in Figure 3B, which shows there is not any significant bias in terms of organelle sensitivity.

Furthermore, these regression models are also adapted to develop more generalized predictive models. These models shall allow researchers to determine the overall effect of test compounds on the cellular integrity and range its application into orphan compounds. Multivariate regression is performed with the independent variable as the organelle SimIdx and dependent variable as all-feature SimIdx. For multivariate regression models (De'Ath, 2002), the independent variables are more than one. For example, the two organelle regression models contain all the possible combinations of two-organelle (as the independent variable) which shall be correlated with the overall cellular response (dependent variable). These models are iterated with all possible combinations of two and three numbers of independent variables. To evaluate the goodness of the novel drug response identification, the model has been implemented to predict data of a large (>1,000) number of new compounds (Supplementary Table 2). Based on the predictions performed and the actual data, the error is computed by evaluating the R squared values. Overall R squared values calculated from the multivariate regression models have been described in Figure 3C. Although all possible combinations in the two and three variable regression models are valuable in making predictions, this accuracy ranking would benefit in understanding the salient organelle that contains signatures to facilitate the predictions. To address this, the organelle combinations that are present in the top 5 accurate models from each of two- and three-variable regression modeling (10 blue colored bars Figure 3C) were taken into consideration. Then the repetitions of each organelle were plotted in Figure 3Di. Out of the 10 cases (totaling 5 × 3 + 5 × 2 = 25 instances of organelles), ER is featured in 8 (32% of 25 is 8) of those. A similar method is taken for the lower 5 models (10 green colored bars in Figure 3C). Then the repetitions of each organelle were plotted in Figure 3Dii. Out of these 10 cases (totaling 25 instances of organelles), Mito is featured in 8 (32% of 25 is 8) of those. Evaluated accuracies from the prediction model affirm ER features are pertinent for the prediction while mitochondria features are lower aptness here. Earlier ER is shown to be high ImpIdx or has more likelihood of being affected upon treatments and Mito's lower aptness (Figure 2D). Overall, along with studying the sensitivity of organelle-specific response, an efficient cellular response prediction model through multivariate regression is developed.

### 3.4. Heterogeneous Cellular Response Mediates Conserved MoA

**Figure 4:**
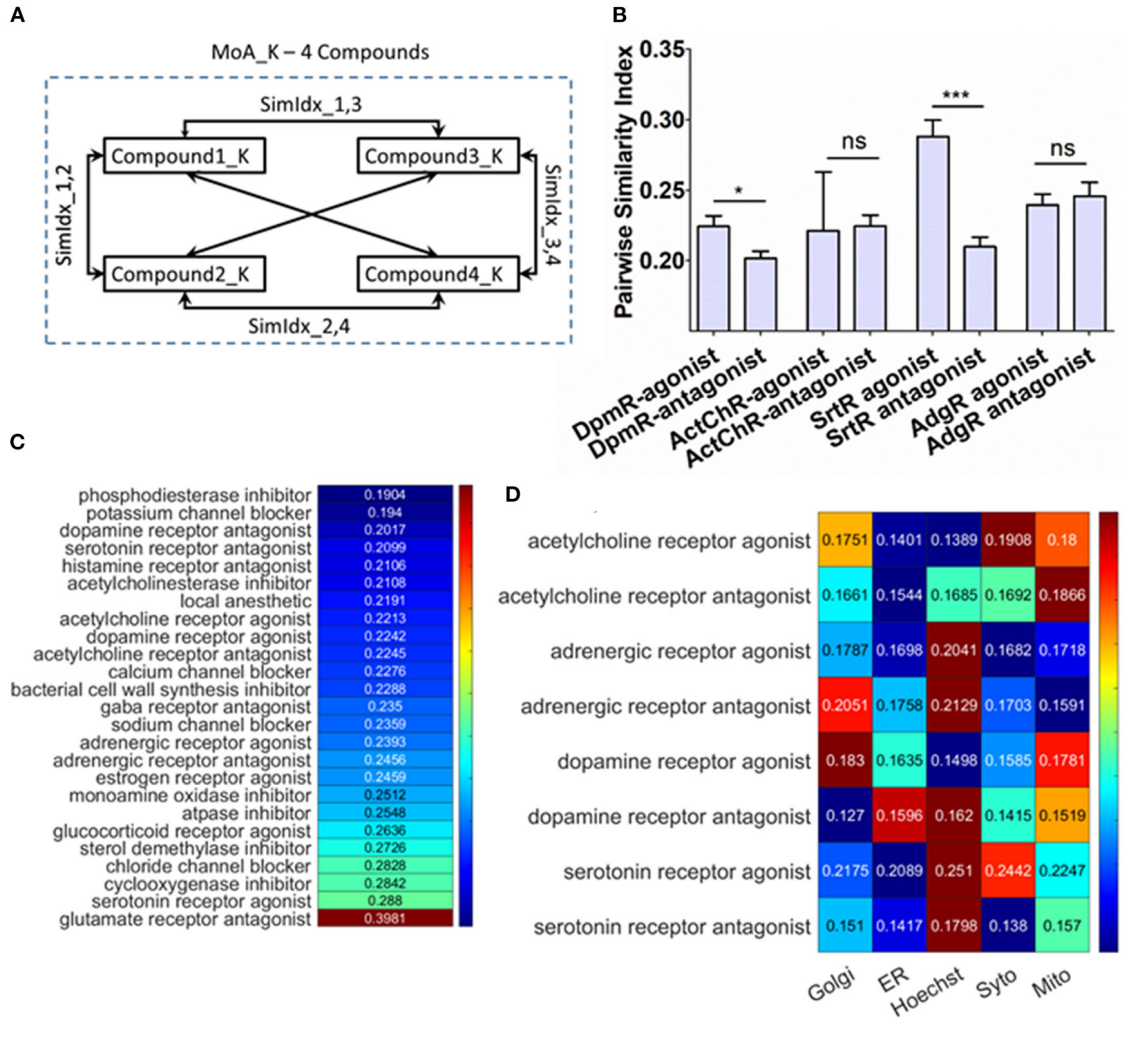
Probing cellular response upon different compound treatment mediating conserved mechanism of action (MoA). **(A)** Schematic of the method to compute the pairwise similarity index between the compounds for the same MoA. **(B)** Pairwise similarity index for antagonist–agonist pair. **(C)** Pairwise similarity index for the MoA enlisted in the dataset, which contains more than three compounds. The colors are column scaled. **(D)** Pairwise similarity index of organelle features specifically for antagonist–agonist pair. The colors are row scaled.

The phenotypic response of cells has now been explored when cells are treated with different signaling modulation treatments. But it would also be interesting to examine the effect on cellular integrity upon treatments with different compounds that enact the same annotated MoA. Since single-cell resolved features can elucidate the heterogeneous response, which can also be used as a biological probe to identify the interactions between cellular machinery. To address this, from the working dataset MoAs were chosen, which were treated with more than three different compounds (Supplementary Table 4) and then pairwise similarity index (PSI) among the compounds is determined as shown in Figure 4A. To generate PSI, SimIdx is determined by checking fraction of similar features between two compounds and then iterated over all possible combinations of compounds (Figure 4A). PSI is similar to SimIdx but is generated by comparing phenotypic profiles between compounds instead of the compound with DMSO. This parameter captured whether the features affected upon these compound treatments are similar (High PSI) or not (Low PSI). If different compounds elicit a similar response that would signify conserved response pathways, which would be captured by higher PSI and vice versa. Interestingly, 24 MoAs are identified for which PSI is <0.3 (Figure 4C). In spite of having conserved MoA, these different compounds exhibit heterogeneity regarding how each of these compounds affects the cellular integrity leading to the low PSI as reported. Thus, it is formulated that different compounds which enacts same outcome (referred here as MoA) might mediate through mechanistically different pathways which enables to evaluate signaling heterogeneity of these MoA cascades.

To probe this furthermore and specifically study how opposing signaling modulations affect the cellular response in terms of the profiled features, the available antagonist and agonist pairs—dopamine receptor (DpmR), acetylcholine receptor (ActChR), serotonine receptor (SrtR), and adregenic receptor (AdgR)—have been chosen. The PSI for these opposing signaling modulations is specifically represented in Figure 4B. The DpmR and SrtR agonist has significantly higher PSI, which might mean the agonist pathways are likely to be more conserved (as across compounds similar features are affected resulting in higher PSI) than respective antagonist ones. But, ActChR and AdgR antagonist–agonist pair shows similar PSI. The overall results show that agonist treatments have at least the same or higher PSI in comparison to their antagonist counterparts.

Subsequently, the effect on organelle integrity is determined by computing the PSI particularly on the organelle features. This indicates the similarity in organelle integrity downstream of the compound treatment (Figure 4A). If an organelle resembles a high PSI, then the compounds have induced similar changes for that organelle. Based on this calculation of the organelle PSI on the agonist–antagonist pair are shown in Figure 4D. This allows inferring that the organelle depicting higher PSI metric are more likely affected (since among the compound treatment this organelle features behaves similarly) upon the respective treatment. In the case of ActChR agonist–antagonist pair, Mitochondria and Syto (nucleolus) features rank as these organelles that get mostly affected through the compounds. Similarly, for AdgR agonist–antagonist pair, Hoechst (Nucleus) and Golgi features are mostly affected through these signaling modulation treatments. Also for the SrtR agonist–antagonist pair, Hoechst (nucleus) is most likely to be affected in both cases followed by Syto (nucleolus) and mitochondria, respectively. In contrast, for DpR the trend reverses. In the case of agonist, Golgi and mitochondria are most likely to be targeted. However, the antagonist treatment targets are different—Hoechst (nucleus) and ER. This observation helps characterize the organelle targets for MoA treatments. For ActChR, AdgR, SrtR agonist–antagonist pair, there is a close resemblance in the most impacted organelle. Since the dopamine receptor affects different targets downstream, this establishes valuable insights regarding signaling plasticity in cancer cells as activation or inactivation of cascades are mediated through different targets. Overall, the quantification helped identify the same MoA inducing treatment could have different downstream targets which hint toward signaling heterogeneity.

## 4. Discussion

In biomedical applications, it is often important to understand the signatures that chemical perturbation imprints on the cell. Quantitative analysis of fluorescent microscopy enables identification of nascent signatures of perturbation (Rohban et al., 2017) as well as health phenotypes (Way et al., 2020). This work is aimed to identify such underlying cellular response signatures by using a publicly available dataset of high content screen. The departure of phenotypic profiles as compared to DMSO as a reference has provided insights regarding the cellular changes induced. The derived metric of SimIdx (as presented in Figures 1A,Bi) from a population of cells is based on the phenotypic impact the signaling modulation treatment causes. SimIdx accredits understanding of phenotypic relationships present in the dataset. This simple yet powerful documentation on diverse data can advance detection of the onset of diseases by labeling the signatures in advance from know datasets.

The subtle changes induced upon treatment are tracked in this study for monitoring phenotypic variations specifically in terms of the organelle. Identification of specific organelle targets could help to target drugs to organelles of maximum relevance. Such a target-directed drug design is critical for maximizing the therapeutic outcome of the drug (Torchilin, 2012). These profiles across various cells aid the identification of novel underlying signatures of organelle-cellular response coupling. Furthermore, the sensitivity analysis of the organelle response (Figure 3B) has shown no particular organelle bias, which could be a result of the transfer of impact from one target to another. The overall cellular behavior is dictated by the rich underlying interacting signaling network. However, hyper-activation (Sever and Brugge, 2015) of signaling cascades is also observed in cancer cells. Hence it is likely that the impact of the treatment on some organelle targets might eventually be relayed onto other organelles (Valm et al., 2017; Cohen et al., 2018). This computational study convenes evidence for signaling hyperactivation, which resonates with the literature hence adds to the validation. These approaches on time-lapse microscopy shall resolve these signatures of cellular response in the temporal domain which enables to probe how the underlying connections evolve with time and develop an organelle interactome.

Based on the deterministic response curves, prediction models have been developed to estimate the overall cellular response by using only specific organelle response features. These models were engineered based on multivariate regression, which is extensively used in engineering analytics (Dumouchel and O'Brien, 1989; Prats-Montalbán et al., 2011). The impact of the treatment on the overall cell is then efficiently predicted based on only fewer organelle stains. For validation, the prediction accuracy is measured on a thousand new candidate compounds (Figure 3C). This prediction ability open avenues to stain cells with a lesser number of fluorescent labels, yet efficiently determine the overall cellular response (Figure 3C) through a simplistic and lesser resource-intensive method. This study also characterizes how ER serves two very critical roles in mediating the cellular response. First, a fraction (80%) of the ER features are affected in at least 240 (out of 270) MoA, making it the most pertinent target organelle (Figure 2D) among the ones tested here. Second, ER also acts as a key organelle (Figure 3Di) in the cell response prediction models. It is known in the literature that ER is also pivotal for cellular homeostasis and extracellular response (Xu et al., 2005; Cao and Kaufman, 2014). Additionally, recent studies have also shown that in cancer ER organelle is stressed and associated signaling pathways are often dysregulated (Yadav et al., 2014; Kato and Nishitoh, 2015; Han and Wan, 2018; Lin et al., 2019). This hints that the ER response is likely to be strongly coupled to the cellular response. Hence, the computational findings in this study align with the earlier reported evidence. Overall, such analysis has paved the way to trace rudimentary trends among organelles.

The PSI, another metric characterized in this study, analysis is applied to examine the differential effect on cellular integrity for the same annotated MoA. Here, the response variability itself has directly been used as a biological probe to access information regarding the functional specificity of these molecular mechanisms. If different compounds elicit a similar response that would signify conserved response pathways, which would be captured by higher PSI and vice versa. This calculation has suitably equipped the study to show that different compound treatments cause differential cellular response yet enacts the conserved final MoA (Figure 4C). Interestingly, this analysis shows how signaling heterogeneity arises by assessing differential impact on the cell caused by similar treatment. For further comparison of cellular response, selective studying of the agonist–antagonist pair has been performed (Figure 4B). This metric has also helped calibrate the trend of organelle (Figure 4D) being affected and gain signaling insights. An understanding regarding the organelle targets for the treatments, which can be beneficial for studying drug targets and their effect. The role of dopamine in mediating neuro-synaptic plasticity is already established (Tecuapetla et al., 2007; Ishikawa et al., 2013; Langlois et al., 2018). Dopamine is also useful in cancer treatment as it results in the shrinking of tumor size (Liu et al., 2019) and inhibiting its progression and exerts anticancer effect (Sarkar et al., 2008; Zhang et al., 2017; Kline et al., 2018). Here at single cell level the interaction between dopamine activation and inactivation with cancer is studied. This revealed that the downstream target switches, which could be a result of rewiring in underlying cascades. Hence, this serves as an elementary evidence for signaling plasticity in cancer cells. Further experimental characterization of this plasticity might reveal the machinery involved as well as advance its role in anticancer therapeutics.

Moreover, with the advent of automation in the cell-painting assay, the screen can be substantially increased enabling to integrate these methods to characterize the downstream effect of a larger number of bioactive compounds. The methods developed here enables integration of high content complex data for studying phenotypic responses and cellular signaling. The report shows how quantitative analysis on cellular imaging screens could be used to derive mechanistic evidence regarding cellular signaling and associated activation, heterogeneity, and plasticity. Identification of these characteristics of molecule treatment will not only enhance understanding of cellular function but also can be applied to transitional research to validate drug and therapeutic effects. This shall also benefit drug discovery and personalized medicine by analyzing subtle changes in the effect of diverse molecules.

In summary, taking advantage of the individual-cell measurements in the high content screen, the cellular phenotypic response has been probed. Subsequently, these facilitated the understanding of varying responses in the downstream effect for multiple treatments on cancer cells, specifically the organelle targets, predicting the overall cellular response efficiently for new candidate molecules and finally evaluate the signaling heterogeneity. Since specifics of the treatment would be identified, this will envisage the identification of hallmarks of both molecular as well as disease targets in cells and open promising avenues through interdisciplinary investigation and quantitative models.

## Data Availability Statement

The dataset used in this study is available from the Broad Bioimage Benchmark Collection public repository. The original contributions presented in the study are included in the article. For additional resources please refer to https://github.com/sayan08/HCA_Target_Response.

## Author Contributions

SB conceptualized and designed the project, developed the computational framework, analyzed the data, interpreted the result, and wrote the manuscript.

## Conflict of Interest

The author declares that the research was conducted in the absence of any commercial or financial relationships that could be construed as a potential conflict of interest.

## Supplementary Material

Supplementary Table 1 List of all MoA used in the study.

Supplementary Table 2 List of the of non-annotated compound BroadIDs.

Supplementary Table 3 List of the profiled features.

Supplementary Table 4 List of MoA where more than three compounds used.

Supplementary Table 5 An example of the dataset (100 × 824). Data from 100 randomly chosen cells. These cells are treated with a compound annotated as adrenergic receptor agonist inducing MoA.

Supplementary Table 6 List of equations used for regression.
